# Green corrosion protection of copper in chloride media with *Calystegia sepium* extract using electrochemical and GC-MS/MS analyses

**DOI:** 10.1038/s41598-026-41526-y

**Published:** 2026-02-27

**Authors:** Mohammad Mahdi Alemnezhad, Mehdi Hosseini, Mohammad Panahimehr

**Affiliations:** 1 Department of Chemistry, Faculty of Basic Sciences, Ayatollah Boroujerdi University, Boroujerd, Iran; 2 Biosensor and Energy Research Center, Ayatollah Boroujerdi University, Boroujerd, Iran

**Keywords:** Green corrosion inhibitor, *Hedge bindweed* extract, Electrochemical impedance spectroscopy, Adsorption thermodynamics, Sustainable corrosion protection, Chemistry, Environmental sciences, Materials science

## Abstract

The use of sustainable and eco-friendly materials for corrosion inhibition has garnered significant attention in recent years. This study investigates the corrosion inhibition performance of *Hedge Bindweed* (*Calystegia sepium*) water extract as a green inhibitor for copper in a neutral saline medium containing 3.5 wt% NaCl. Electrochemical techniques, including potentiodynamic polarization and electrochemical impedance spectroscopy (EIS), were employed to evaluate the inhibition behavior of the extract over a temperature range of 25–65 °C. The results revealed a maximum inhibition efficiency of 91.9% at an extract concentration of 5 × 10^− 3^ M. Weight loss measurements further confirmed the effectiveness of the inhibitor, yielding an inhibition efficiency of 90.4%. Notably, even at elevated temperatures, the extract maintained satisfactory performance, retaining approximately 50% inhibition efficiency at 65 °C, indicating reasonable thermal stability. Temperature-dependent linear sweep voltammetry (LSV) measurements provided insight into the thermodynamic and kinetic aspects of the corrosion process, showing an increase in activation energy in the presence of the inhibitor, consistent with a hindered corrosion reaction. Surface morphology and elemental composition analyses using scanning electron microscopy (SEM) and energy-dispersive X-ray spectroscopy (EDS) confirmed the formation of a protective layer on the copper surface. Furthermore, gas chromatography–mass spectrometry (GC-MS/MS) analysis identified several bioactive compounds responsible for the inhibition effect. Thermodynamic adsorption studies demonstrated that the inhibitor adsorption followed the Langmuir isotherm, suggesting a predominantly physisorption-controlled mechanism. Overall, these findings highlight *Hedge Bindweed* extract as a highly efficient, sustainable, and eco-friendly corrosion inhibitor for copper in saline environments.

**Supplementary Information:**

The online version contains supplementary material available at 10.1038/s41598-026-41526-y.

## Introduction

Corrosion is a naturally occurring process that severely affects the durability and service life of metallic structures, resulting in substantial economic losses^1^. Corrosion-related material degradation accounts for approximately 3–4% of a country’s gross domestic product (GDP)^2^, corresponding to an estimated annual cost of nearly 2.5 trillion USD worldwide^3^. In industrial applications such as heat exchangers, power plant cooling systems, and copper smelting facilities, corrosion remains a major challenge, directly impacting productivity, operational efficiency, and equipment reliability^4^. Beyond economic considerations, metal corrosion also poses serious safety risks by degrading mechanical properties, weakening structural integrity, and increasing the probability of catastrophic failures, including fires and explosions^5^.

Copper and its alloys are extensively used across a wide range of industries due to their excellent electrical and thermal conductivity, high machinability^6^, and strong resistance to electromigration^7^. These advantageous properties make copper indispensable for applications such as electrical wiring, heat exchangers, and fluid transport pipelines^8^. Although copper naturally forms a protective oxide layer upon exposure to atmospheric conditions, this passive film becomes unstable in aggressive environments, rendering the metal susceptible to corrosion and leading to significant economic and operational challenges^9^. While copper exhibits relatively high corrosion resistance due to the formation of a surface oxide layer, its degradation mechanisms strongly depend on the chemical nature of the surrounding environment. In acidic media such as HCl^10,11^ and H_2_SO_4_, copper corrosion is predominantly driven by solution acidity, where proton reduction and acid-assisted dissolution control the corrosion kinetics. In such systems, aggressive anions mainly influence corrosion by stabilizing soluble copper complexes and accelerating metal dissolution^12–15^.

In contrast, in neutral or near-neutral chloride-containing environments, such as NaCl solutions, corrosion is governed primarily by the specific adsorption of chloride ions rather than by proton activity. Chloride ions disrupt the protective oxide layer on copper surfaces, promote the formation of unstable Cu-Cl complexes, and facilitate localized corrosion phenomena such as pitting. This anion-controlled mechanism is particularly relevant to marine, cooling-water, and saline industrial environments, where copper components are commonly exposed to high chloride concentrations under non-acidic conditions^16–18^.

Although copper exhibits intrinsic corrosion resistance, it undergoes degradation in the presence of strong oxidizing species, including sulfates, chlorides, and oxides^19,20^. In many industrial operations, particularly copper pickling processes, sulfuric and hydrochloric acids are extensively employed to eliminate surface oxides, thereby accelerating copper dissolution and increasing material loss. Likewise, in microelectronics fabrication, chemical mechanical polishing (CMP) relies on complexing agents to promote copper removal, which can significantly increase corrosion susceptibility if not carefully regulated^21,22^. Under severely aggressive environments, such as physiological media containing high chloride levels, copper becomes highly susceptible to localized pitting corrosion, which critically compromises its long-term stability and reliability^23,24^.

To effectively control copper corrosion, several preventive approaches have been proposed. Alloying strategies, involving the incorporation of other metals, improve corrosion resistance by enhancing structural strength and lowering chemical reactivity toward aggressive ions. Surface protection methods, including organic coatings and polymer-based barrier films, function as physical shields that restrict direct interaction with corrosive species^25,26^. Nevertheless, among the most efficient and commonly implemented techniques is the application of organic corrosion inhibitors^27,28^.

Organic corrosion inhibitors suppress copper degradation by adsorbing onto the metal surface and forming a compact protective film that limits direct exposure to corrosive agents^29^. Their inhibitory performance is strongly influenced by molecular architecture, particularly the presence of aromatic systems and heteroatoms bearing lone pair electrons, which promote robust adsorption through both physical and chemical interactions. In acidic media, functional groups such as -COOH, -OH, -SH, and -NH_2_^30^ significantly enhance inhibition efficiency by reinforcing the interfacial bonding between inhibitor molecules and the copper substrate^31^. Owing to their abundant availability, low cost, and high protective efficiency, organic inhibitors remain among the most practical and widely utilized solutions for mitigating copper corrosion in industrial as well as environmental applications^32^.

*Hedge bindweed* (*Calystegia sepium* (L.) R. Br.) is a highly persistent and invasive plant species that presents serious challenges in agricultural systems worldwide. Together with field bindweed, it is considered one of the most troublesome weeds, characterized by vigorous growth, an extensive root network, and intense competition with crops for essential nutrients and water. Its rapid proliferation leads to reduced crop yields and interferes with harvesting operations, imposing substantial economic losses on cereal, vegetable, and fruit production. Moreover, *hedge bindweed* acts as a reservoir for insects and an alternative host for plant pathogens, further intensifying agricultural damage. Despite numerous control strategies, including mechanical and chemical methods, complete eradication remains elusive, with most approaches offering only temporary suppression^33^. Conventionally viewed solely as an agricultural pest due to its invasive nature and lack of economic value, *hedge bindweed* can be strategically repurposed as a green corrosion inhibitor, offering a novel and sustainable pathway for resource utilization. The extraction of bioactive phytochemicals from this weed and their application in metal corrosion protection transforms an environmental burden into an eco-friendly industrial solution. This approach not only reduces reliance on synthetic inhibitors but also supports weed management by assigning a beneficial application to hedge bindweed^34^.

In recent years, the development of green corrosion inhibitors has attracted considerable interest within the corrosion science community due to their environmental compatibility and economic advantages, including value addition from natural resources. A wide range of plant-derived inhibitors, such as *Rhododendron simsii*, *Pyracantha fortuneana*, lemonal terpenoids, and honeysuckle, have been systematically investigated^35–39^. In the present work, for the first time, the corrosion inhibition potential of *Hedge Bindweed* Extract (HBE) for copper in a 3.5 wt% NaCl medium is systematically evaluated. The protective performance of HBE is examined using electrochemical techniques, including potentiodynamic polarization and electrochemical impedance spectroscopy (EIS), in conjunction with gravimetric weight loss measurements. Additionally, the findings are supported by comprehensive surface and chemical analyses, including gas chromatography-mass spectrometry (GC-MS), scanning electron microscopy (SEM), energy-dispersive X-ray spectroscopy (EDS), and thermodynamic evaluation, providing in-depth insight into the inhibition mechanism and overall efficiency of HBE.

This study represents the first comprehensive investigation into the corrosion inhibition performance of *Calystegia sepium* extract for copper in chloride-rich saline environments, addressing a critical gap in the literature where previous studies have largely focused on steel substrates or synthetic inhibitors. The originality of this work lies in the integration of electrochemical techniques, thermodynamic and adsorption analyses, surface characterization, and detailed GC–MS/MS phytochemical profiling within a single experimental framework. This combined approach enables a direct correlation between the molecular constituents of the plant extract and its adsorption-driven inhibition behavior on copper surfaces. Moreover, the mechanistic insights gained from the electrochemical–thermodynamic interpretation contribute to a deeper understanding of green, plant-based corrosion inhibitors in aggressive saline media, thereby reinforcing the scientific relevance and innovation of the present study.

## Experimental

### Materials and solvents

The Calystegia sepium (*Hedge Bindweed*) plant material used in this study was collected from the green area within Ayatollah Boroujerdi University (Boroujerd, Lorestan Province, Iran), with official permission granted by the university authorities. A voucher specimen was prepared and deposited in the Herbarium of the Chemistry Research Laboratory, Department of Chemistry, Ayatollah Boroujerdi University, under voucher number AARU-Herb-CS-2025, where it is publicly accessible upon request. The sodium chloride salt and ethanol used in this study were of analytical grade and supplied by Merck, whereas the acetone used for washing purposes was of industrial grade and procured from Metron (Middle East Company, Dubai, UAE). The copper specimens employed in this investigation consisted of rectangular copper sheets with dimensions of 1 × 3 cm and a thickness of 1 mm. This copper electrodes were fabricated from commercial copper sheet with high purity, as confirmed by quantometric analysis. The material consisted predominantly of copper (≈ 99.8 wt%), with trace amounts of common metallic impurities (such as Si, Ni, Pb, S and Bi) below 0.2 wt% in total (UNS C16400 alloy), indicating suitability for corrosion and electrochemical investigations.

### Instruments

An electric grinder (Dessini BT-601 S) was used, and GC-MS/MS analysis was carried out using an Agilent GC 7890 A Triple Quadrupole MS 7000 system to identify the major and effective compounds in the *Hedge Bindweed* Extract (HBE) employed as corrosion inhibitors for copper in a 3.5 wt% NaCl solution.

Potentiodynamic polarization tests were conducted using a potentiostat/galvanostat operated in linear sweep voltammetry (LSV). The EIS experiments were performed using a PGSTAT 204 N potentiostat/galvanostat (Metrohm Autolab, Echo Chemie B.V., Netherlands). SEM-EDS (TESCAN, VEGA/II.) analysis was employed to examine the surface morphology of the electrodes and the elemental composition of the copper electrode surface.

GC-MS/MS analysis was carried out using an Agilent GC 7890 A system coupled with a Triple Quadrupole MS 7000 detector, equipped with an HP-5MS capillary column (0.25 μm film thickness). The oven temperature program was set to 45 °C (held for 1 min), followed by a heating rate of 2 °C. min^− 1^ up to 200 °C, where it was maintained for 30 min. Helium was used as the carrier gas at a constant flow rate. The resulting chromatograms were analyzed to identify the main bioactive compounds present in the *Hedge Bindweed* extract.

### Plant material processing and extract preparation

The collected plant was washed twice with tap water and once with distilled water. Subsequently, it was chopped into smaller pieces and dried in an oven at 50 °C for 24 h. Once fully dried, the plant material was ground into a homogeneous powder using an electric grinder and sieved through a 40-mesh sieve to ensure uniform particle size. The resulting powder was stored in a sealed container in a cool and dry place for subsequent experimental procedures.

The *Hedge bindweed* extract (HBE) was prepared using two solvents: water and ethanol. The aqueous extract was prepared by placing 2 g of the sieved plant powder in 50 mL of distilled water, followed by ultrasonication at 70 °C for 12 h. After filtration, the extract was used at concentrations of 5, 10, 15, and 20 v/v% in a 3.5 wt% NaCl solution. The ethanolic extract was prepared by placing 4 g of plant powder in a Soxhlet extractor, using 100 mL of analytical-grade ethanol for 24 h. The resulting ethanolic extract was subjected to GC-MS analysis to identify the major components and active ingredients present^40^.

The aqueous extract was used for all corrosion inhibition experiments, whereas the ethanolic extract was employed exclusively for GC-MS/MS analysis due to its higher efficiency in extracting semi-volatile organic compounds, allowing reliable compound identification. It should be noted that the ethanolic extract itself cannot be considered a corrosion inhibitor because ethanol may contribute to corrosion inhibition, and its effects are not representative of the extract’s intrinsic activity. Moreover, ethanol is an organic solvent, whereas the focus of this study is on the aqueous extract, which is readily accessible, environmentally friendly, and suitable for sustainable corrosion protection. This distinction and the limitation that the GC-MS/MS-identified compounds may not fully represent the composition of the aqueous extract have been explicitly clarified in the revised manuscript. The plant material (*Hedge bindweed*) used in this study was formally identified and authenticated by the corresponding author, Dr. Mehdi Hosseini, in accordance with the official permission issued by the Ayatollah Boroujerdi University.

### Copper electrode preparation

The copper samples were polished sequentially with P600, P1200, and P2000 sandpaper to remove any existing oxide or rust layers. To prevent surface contamination, the polished electrodes were washed with distilled water, followed by acetone, and then rinsed again with distilled water. To ensure high reproducibility and measurement accuracy, the copper electrodes were masked using a metallic template, leaving an exposed geometric surface area of 0.38 cm^2^. This approach not only minimized edge effects and non-uniform current distribution but also prevented overvoltage issues that could occur at larger electrode areas due to equipment limitations. All electrochemical parameters, including corrosion current density (*j*_*corr*_), corrosion potential (*E*_*corr*_), and charge transfer resistance (*R*_*ct*_), were calculated by normalizing the measured currents to the actual exposed surface area. Consequently, the reported values are area-independent and fully comparable with standard corrosion studies reported in the literature.

### LSV and EIS assessment of HBE as corrosion inhibitor

To evaluate the efficiency of HBE in mitigating the corrosion of copper in a 3.5 wt% NaCl solution, potentiodynamic polarization tests were performed using a potentiostat/galvanostat operated in linear sweep voltammetry (LSV) mode, along with electrochemical impedance spectroscopy (EIS) measurements. LSV measurements were carried out at a scan rate of 5 mV. s^− 1^ within a potential range of − 250 to + 250 mV with respect to the stabilized OCP.

The EIS experiments were performed using a potentiostat/galvanostat. All electrochemical measurements were executed in a conventional three-electrode setup comprising an Ag/AgCl reference electrode, a platinum counter electrode, and copper specimens acting as the working electrode. EIS spectra were recorded over a frequency range of 0.1 to 10^5^ Hz around the open-circuit potential (OCP) of the system by applying a sinusoidal AC perturbation of 10 mV. Prior to all electrochemical measurements, the copper working electrode was immersed in the test solution and allowed to stabilize at open-circuit potential (OCP) for 30 min, until the potential variation was less than ± 2 mV over a 5 min period, indicating a stable electrochemical equilibrium.

The inhibition efficiencies (*IE%*) determined from LSV and EIS were computed using Eqs. (1) and (2), respectively. In these equations, jcorr and *R*_*ct*_ indicate the corrosion current density and charge transfer resistance in the presence of HBE, respectively, while *j*^*⁰*^_*corr*_ and *R*^*⁰*^_*ct*_ denote the corresponding values in the absence of HBE^3^.

1 $$\:IE\left(\mathrm{\%}\right)=\left(1-\frac{{j}_{corr}}{{j}_{corr}^{^\circ\:}}\right)\times\:100$$

2 $$\:IE\left(\mathrm{\%}\right)=\left(1-\frac{{R}_{ct}^{^\circ\:}}{{R}_{ct}}\right)\times\:100\:\:\:\:\:\:\:\:\:\:$$

## Results and discussion

### GC-MS/MS analysis for HBE

The results are summarized in Table 1 and Fig. S1. As illustrated, all identified compounds in the plant extract feature at least one heteroatom- nitrogen (N), oxygen (O), or sulfur (S). These heteroatoms harbor lone electron pairs capable of interacting with the vacant orbitals of copper atoms. This interaction is considered the primary mechanism underlying the corrosion inhibition behavior observed, as further elaborated in the subsequent sections.

GC-MS/MS analysis of the ethanolic *Hedge Bindweed* extract revealed six major compounds with relatively high abundances, while several additional components were detected only at trace levels. The relative abundance of each major compound was estimated based on normalized GC peak areas and is expressed as a percentage of the total detected extract.

Among the identified compounds, 2,2-nitroethanol and 5,2-hydroxypropionamide exhibited the highest relative abundances, followed by 3-bis(2-aminoethyl) amine and 2-ethyl-2-oxopropyl sulfide. Compounds such as 3-pentanethiol and 2.3,3-Dimethyldihydro-2(3 H)-furanone were present in lower but still detectable amounts. Minor unidentified constituents were observed with very low peak intensities (< 5% individually) and were therefore not considered dominant contributors to the inhibition mechanism.

**Table 1:** Identification of active ingredients in the ethanolic extract of *Hedge Bindweed* (*Calystegia sepium*) through GC-MS/MS analysis.

Number	1	2	3	4	5	6
**Compound name**	5.2-hydroxypropionamide	2.2-Nitroethanol	3-Bis(2-aminoethyl) amine	2-Ethyl 2-oxopropyl sulfide	3-Pentanethiol	2.3,3-Dimethyldihydro-2(3 H)-furanone
**Formula**	C_3_H_7_NO_2_	C_2_H_5_NO_3_	C_4_H_13_N_3_	C_5_H_10_OS	C_5_H_12_S	C_6_H_10_O_2_
**Structure**						
**Percentage (%)**	32.09	50.08	9.61	3.31	3.08	1.83

### Potentiodynamic polarization analysis of HBE: Efficiency and thermodynamic control

Potentiodynamic polarization analysis was utilized to obtain key electrochemical parameters, including the corrosion potential (*E*_*corr*_), corrosion current density (*j*_*corr*_), and the anodic and cathodic Tafel slopes. The corresponding data derived from Fig. 3a are presented in Table 2.

As evident from the table, increasing the concentration of the inhibitor results in higher inhibition efficiency and lower corrosion rate. Moreover, Fig. 3a demonstrates that with increasing inhibitor concentration, the Tafel plots shift toward more positive potentials, indicating an increase in Ecorr and suggesting improved thermodynamic control over the copper corrosion process in the NaCl solution. Simultaneously, a decrease in jcorr is noted, shifting toward lower values, which reflects the suppressed kinetics of the corrosion reaction and confirms the effective performance of HBE as a corrosion inhibitor. At an HBE concentration of 20 v/v%, a maximum inhibition efficiency of 91.9% was attained. However, since the efficiency at 15 v/v% was also high (87.2%), this lower concentration can be regarded as optimal to reduce inhibitor consumption while maintaining substantial protection.

**Table 2:** Data extracted from LSV curves at different concentrations of HBE in a 3.5 wt% NaCl solution at room temperature.

HBE Conc. (v/v%)	E_corr_ (mV. cm^−2^)	j_corr_ (µA.cm^−2^)	b_c_ (mV. dec^− 1^)	b_a_ (mV. dec^− 1^)	IE (%)
0	−807.6	17.2	543.6	196.9	-
5	−685.9	7.3	397.6	81.8	57.6
10	−681.3	5.1	346.8	80.4	70.3
15	−541.6	2.2	276.7	94.4	87.2
20	−535.3	1.4	230.5	88.0	91.9

The potentiodynamic polarization curves reveal that the addition of HBE affects both the anodic dissolution and cathodic reduction reactions of copper in NaCl solution. The displacement of the corrosion potential (*E*_*corr*_) relative to the uninhibited system remains below ± 85 mV for all inhibitor concentrations, indicating that HBE can be classified as a mixed-type inhibitor.

Nevertheless, at higher HBE concentrations, a more pronounced suppression of the anodic branch is observed, as evidenced by the greater anodic shift and the stronger reduction in anodic current density. This behavior suggests that, while HBE acts as a mixed-type inhibitor, its inhibitory action becomes anodically predominant at elevated concentrations^41^.

As depicted in Fig. 3b, the shift in corrosion potential between the blank and 10 v/v% HBE is less than 85 mV, and the corrosion potential variations at higher concentrations do not exceed this range. In this mix-type inhibitor, the copper corrosion process proceeds via both anodic and cathodic pathways in the presence of the inhibitor. Consequently, the following equations can be formulated for the anodic (Eq. 4 to 7)^42,43^ and cathodic (Eq. 3) reactions of the copper corrosion process in the presence of HBE. As seen in the anodic reaction series (Eqs. 4–7), first, chlorine anions attack the copper surface, causing copper metal to dissolve as Cu+. Then, Cu^+^ interacts with chloride ions in the corrosive solution, leading to the formation of [CuCl]^−^ precipitate. [CuCl]^−^ is highly reactive with Cl^−^, resulting in the formation of the unstable [CuCl_2_]^−^ complex. Finally, [CuCl_2_]^−^ undergoes decomposition in the solution, forming the final corrosion product, Cu^2+^.

3 $${O_2}\, + \,2{H_2}O\, + \,4\overline{e} \to 4O{H^ - }$$

4 $$C{u_{(s)}}\;\xrightarrow{{C{l^ - }}}C{u^ + }_{(aq)} + \overline{e}$$

5 $$C{u^ + }_{(aq)}\, + \,C{l^ - }_{\left( {aq} \right)} \to CuC{l_{(insoluble)}} + \overline{e}$$

6 $$CuC{l_{(insoluble)}}\, + \,C{l^ - }_{\left( {aq} \right)} \to {\left[ {CuC{l_2}} \right]^ - }_{\left( {aq} \right)}$$

7 $${\left[ {CuC{l_2}} \right]^ - }_{\left( {aq} \right)} \to C{u^{2 + }}_{(aq)}\, + \,2C{l^ - }_{\left( {aq} \right)} + \overline{e}$$

**Fig. 1:**
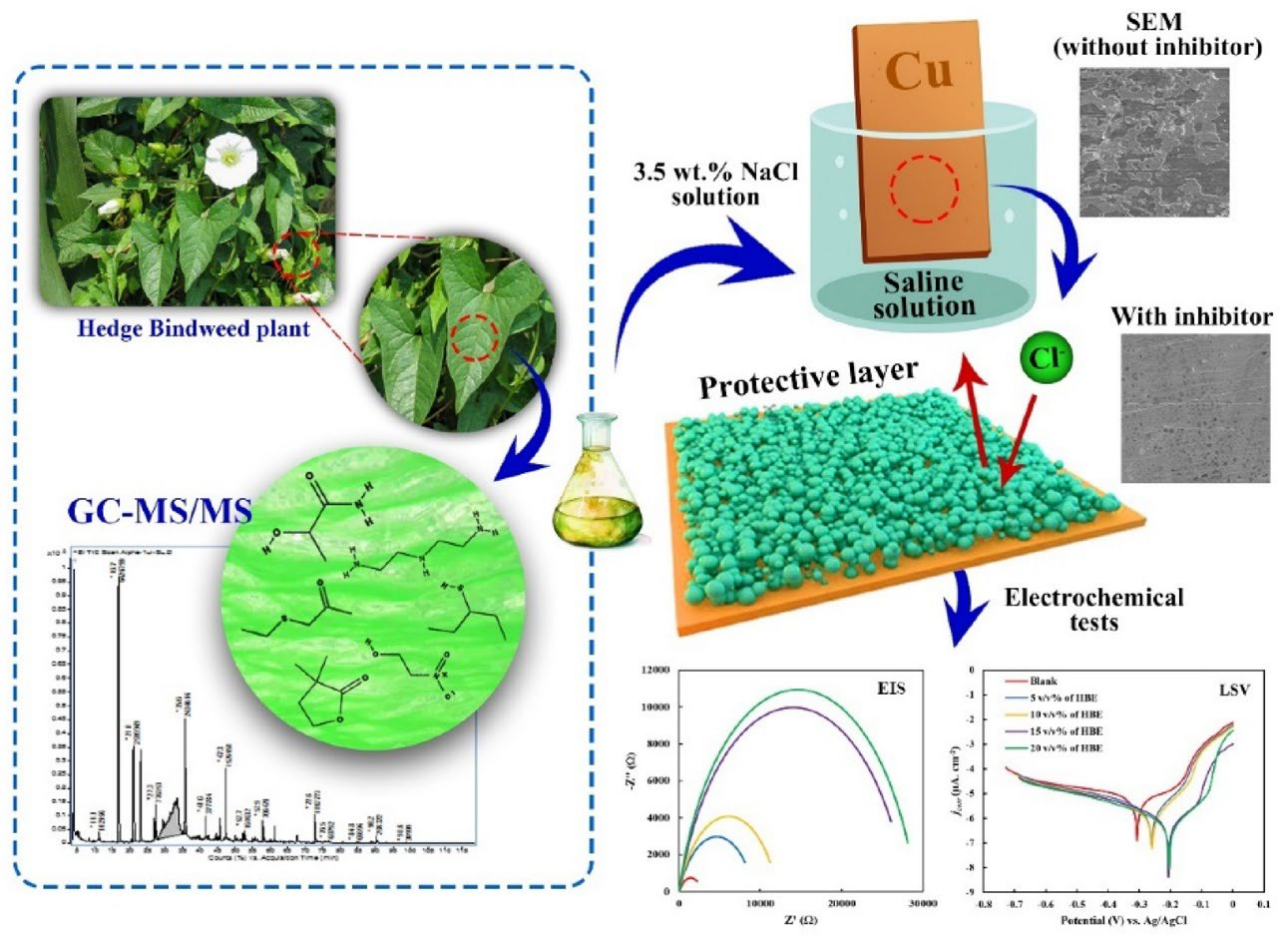
Schematic representation of copper corrosion inhibition in 3.5 wt% NaCl solution by *Calystegia sepium* extract through adsorption-induced protective layer formation.

The schematic representation outlines the overall inhibition mechanism of the Calystegia sepium extract in a saline environment (Fig. 1). In the absence of the inhibitor, aggressive chloride ions facilitate copper dissolution through anodic oxidation accompanied by the corresponding cathodic reactions. Upon addition of the extract, bioactive organic constituents identified by GC-MS/MS adsorb onto the copper surface, leading to the formation of a protective adsorption layer. This layer acts as a physical barrier that restricts the access of chloride ions to active surface sites, thereby suppressing charge transfer processes and reducing the overall corrosion rate. The proposed mechanism is fully consistent with the electrochemical responses obtained from polarization and impedance measurements.

### Electrochemical impedance spectroscopy (EIS) evaluation of HBE inhibition efficiency

**Fig. 2:**
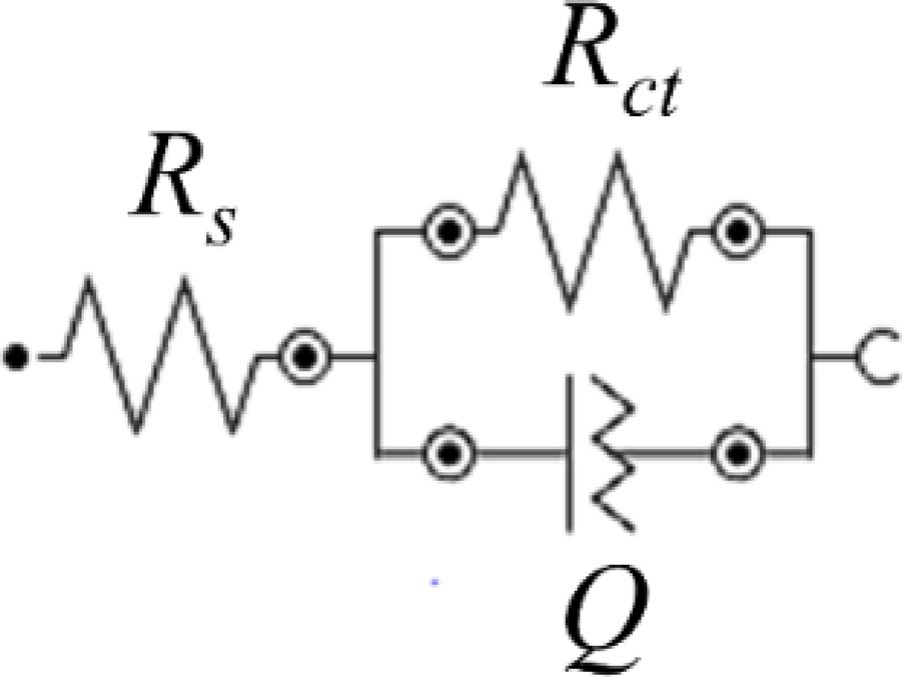
Equivalent electrical circuit *R*_*s*_(*R*_*ct*_*Q*) used to fit the EIS data, where *R*_*s*_ represents the solution resistance, *R*_*ct*_ the charge-transfer resistance, and *Q* the constant phase element associated with the non-ideal double-layer capacitance.

**Fig. 3:**
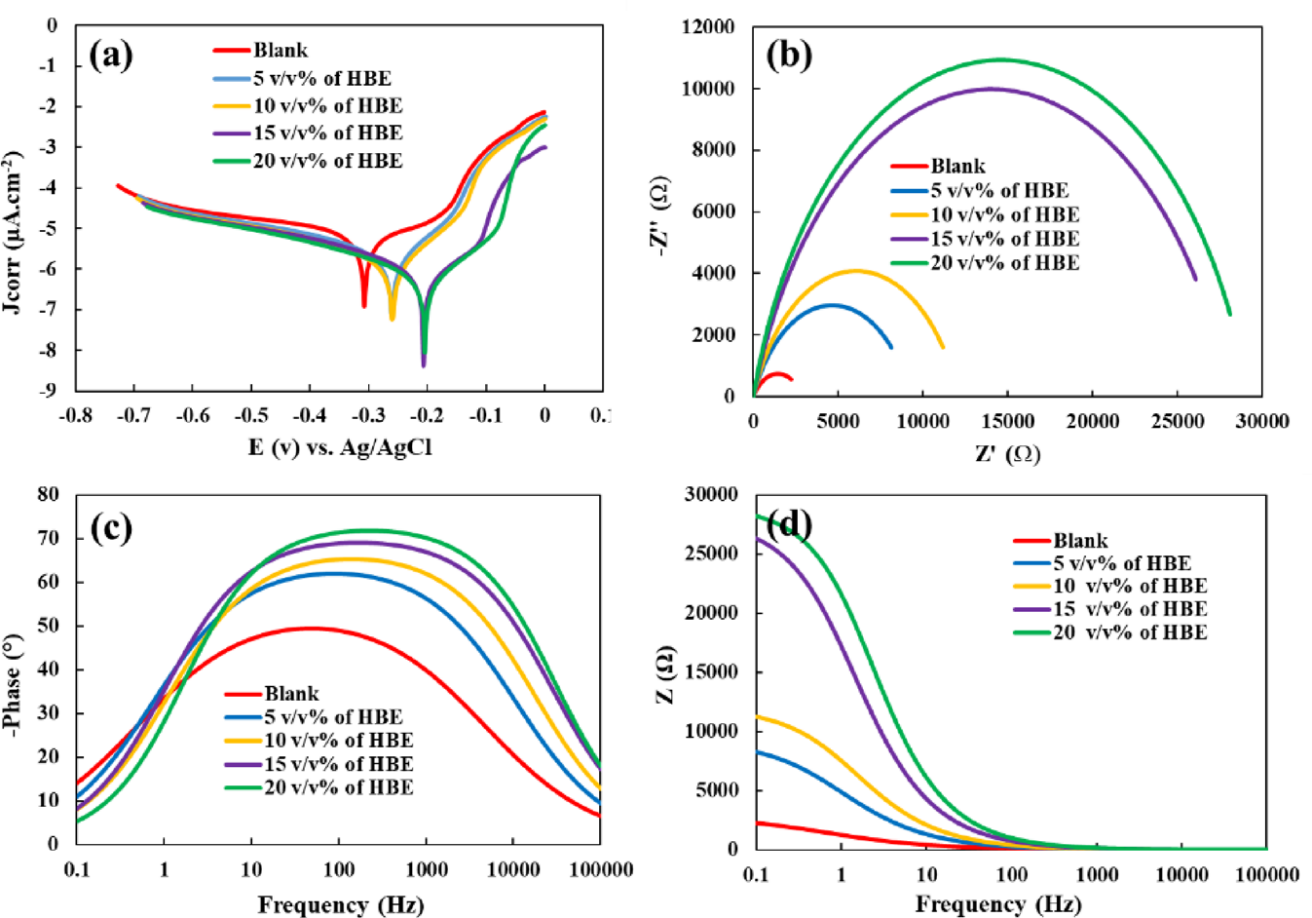
**(a)** Linear sweep voltammograms (LSV, Tafel plots) for different concentrations of HBE in a 3.5 wt% NaCl solution, **(b)** Nyquist, **(c)** Bode phase, and **(d)** Bode magnitude graphs of Cu corrosion in 3.5 wt% NaCl solution in the absence and presence of varying concentrations of HBE.

One of the important electrochemical tests for investigating metal corrosion is electrochemical impedance spectroscopy (EIS) analysis. Figure 3b-d shows the impedance diagram obtained from the Cu electrode in a 3.5 wt% NaCl corrosive solution with and without different concentrations of the HBE inhibitor. As seen from the semicircles in Fig. 3b-d, the data from this analysis follow the equivalent circuit of *R*_*s*_(*R*_*ct*_*Q*). *R*_*s*_ and *R*_*ct*_ represent the solution resistance and the charge transfer resistance at the active surface of the Cu electrode, respectively. Additionally, when the copper electrode is immersed in a solution containing the inhibitor, the HBE molecules form a non-uniform protective layer, resulting in the formation of a non-ideal double-layer capacitor (constant phase element, CPE or *Q*) (Fig. 2).

The capacitance of the CPE can be calculated using Eq. 8. The double-layer capacitance (*C*_*dl*_) reflects the capacitive behavior of the copper-electrolyte interface and provides insight into surface modifications occurring during corrosion inhibition. In the absence of HBE, the relatively high *C*_*dl*_ value indicates a bare copper surface in direct contact with the electrolyte. As the HBE concentration increases, a gradual decrease in *C*_*dl*_ is observed (Table 3), which can be attributed to the adsorption of inhibitor molecules onto the copper surface. This adsorption process leads to either a reduction in the local dielectric constant at the interface and/or an increase in the effective thickness of the electrical double layer, consistent with the formation of a protective adsorbed film. The observed trend in *C*_*dl*_ supports the inhibition mechanism inferred from polarization measurements and confirms the protective role of HBE in the NaCl medium.

Moreover, the data obtained from the electrochemical fits of the impedance diagrams (Table 4) indicate that the inhibition efficiency (*IE%*) of this inhibitor is very similar to that derived from the LSV analysis, further validating the reliability of the test. In the above equation, fmax and Y_0_ denote the frequency of the maximum impedance imaginary component and the magnitude of the CPE, respectively. The parameter *n* is an adjustable factor, given by n = α/(π/2) where α represents the phase angle.

8 $${C_{dl}} = {\text{ }}{Y_0}{(2\pi{f_{max}})^n}^{ - 1}$$

The Nyquist plots exhibit depressed semicircles rather than ideal capacitive loops, indicating non-ideal interfacial behavior. This deviation from ideality is commonly associated with surface roughness, inhomogeneous adsorption of inhibitor molecules, and a distribution of relaxation times at the copper-electrolyte interface. To account for this non-ideal behavior, a constant phase element (*Q*) was used instead of an ideal capacitor in the equivalent circuit.

The employed equivalent circuit, *R*_*s*_(*R*_*ct*_*Q*), assumes a single dominant time constant. In the presence of HBE, the time constant related to the adsorbed inhibitor layer may partially overlap with that of the charge-transfer process, making it difficult to resolve them as separate contributions within the experimental frequency window. Therefore, the use of a single *R*_*ct*_*-Q* element provides a physically reasonable and statistically reliable fit to the EIS data.

Additional insight into the corrosion inhibition behavior of HBE is provided by the Bode plots shown in Fig. 3c-d. The Bode magnitude plots reveal a clear increase in the impedance modulus (*Z*) at low frequencies with increasing HBE concentration, reflecting an enhancement in charge-transfer resistance and, consequently, improved corrosion protection.

The corresponding phase angle plots exhibit a broader and more pronounced maximum in the mid-frequency range upon addition of HBE. This increase in phase angle indicates a more capacitive response of the copper-electrolyte interface, consistent with the adsorption of inhibitor molecules and the formation of a protective surface film. The absence of additional phase angle maxima further supports the presence of a single dominant time constant, in agreement with the equivalent circuit model used for fitting the impedance data.

**Table 3:** Electrochemical parameters obtained from the fitted Nyquist plots for the corrosion of copper in 3.5 wt% NaCl solution with and without the HBE inhibitor at room temperature.

Inhibitor Conc.	*R*_s_ (Ω. cm^2^)	*R*_ct_ (kΩ. cm^2^)	CPE (µMho. s ^*n*^)	C_dl_ (µF. cm^2^)	*n*	IE%
0	10.7	2.9	175.0	113.5	0.6	-
5	9.0	9.3	35.4	22.8	0.7	69.3
10	8.1	12.1	19.2	12.0	0.8	76.4
15	8.8	28.0	8.4	5.7	0.8	89.8
20	9.4	29.2	5.1	3.3	0.8	90.2

### Weight loss analysis

Weight loss analysis is a standard non-electrochemical method commonly employed to determine the corrosion rate and evaluate the inhibition performance of various compounds in corrosive environments. In this study, five copper specimens were mechanically polished, thoroughly rinsed, and accurately weighed prior to immersion. These specimens were then immersed in a 3.5 wt% NaCl solution in the absence and presence of various concentrations of the HBE inhibitor for 72 h. After the immersion period, the corrosion inhibition efficiency (*IE%*) of HBE was calculated from the measured weight loss of the copper specimens using Eq. (9).

9 $$\:IE\%=\:\frac{{v}_{0}-v}{{v}_{0}}\times\:100\%$$

In the above equation, *v* and *v*_*₀*_ represent the corrosion rates in the presence and absence of the HBE inhibitor, respectively, which are calculated using Eq. (10), where *W*_*₀*_ and *W* are the weights of the electrode before and after immersion, *S* is the surface area of the electrode, and *t* is the exposure time (h). The results obtained from the weight loss analysis are presented in Table 4. These data indicate that, with increasing inhibitor concentration, the corrosion rate progressively decreases, while the inhibition efficiency (*IE%*) correspondingly increases. The close agreement between these results and those obtained from the electrochemical analyses further supports the reliability and consistency of the obtained findings.

10 $$\:v=\:\frac{{w}_{0}-w}{S.t}$$

**Table 4:** Weight loss analysis data for the Cu electrode in a 3.5 wt% NaCl corrosive solution in the presence and absence of various concentrations of HBE at room temperature.

HBE concentration (v/v%)	v (g. m^−2^. h^−1^)	IE%
Blank	3.8	-
5	1.5	60.1
10	0.9	77.4
15	0.6	84.8
20	0.4	90.4

### SEM-EDS analysis of corrosion damage and HBE protective performance

**Fig. 4:**
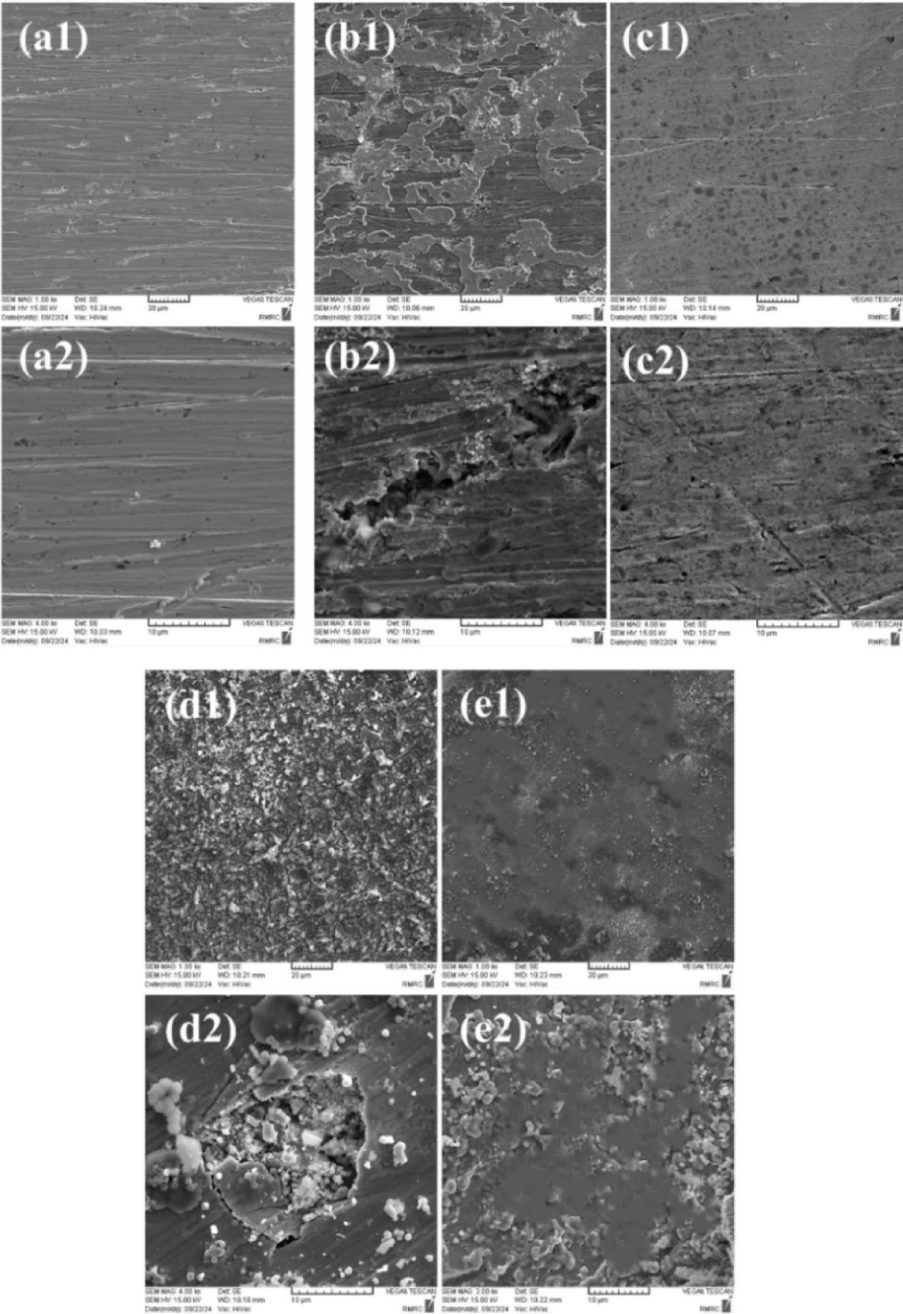
SEM images acquired from the control copper electrode **(a)**, the electrode immersed in the corrosive solution for 1 h without **(b)** and with **(c)** the inhibitor solution, and copper electrodes immersed for 24 h in the corrosive solution of 3.5 wt% NaCl in the presence **(e)** and absence **(d)** of HBE.

SEM-EDS analysis was employed to examine the surface morphology of the electrodes and the elemental composition of the Cu electrode surface. For this purpose, five Cu electrodes were polished and prepared, one of which was considered a control electrode (Fig. 4a), and the surface changes of the remaining four electrodes after 1 and 24 h in the presence (Fig. 4c, e) and absence (Fig. 4b, d) of HBE in a corrosive solution of 3.5 wt% NaCl were investigated. As seen in Fig. 4, the corrosion rate in the corrosive solution progresses with time, and the black spots that indicate corrosion-induced defects increase. However, Fig. 4e demonstrates that using the inhibitor solution, even after 24 h of exposure to the corrosive solution, results in a sharp reduction in corrosion effects, confirming the results obtained from electrochemical and weight loss analyses. The notable observation regarding the presence of some minor defects on the surface of the copper electrode immersed in the corrosive solution for 24 h in the presence of the inhibitor (Fig. 4e) is that these surface shows limited and sparse defects compared to the control sample, which indicates that the efficiency of the inhibitor has decreased after 24 h but remains effective.

**Fig. 5:**
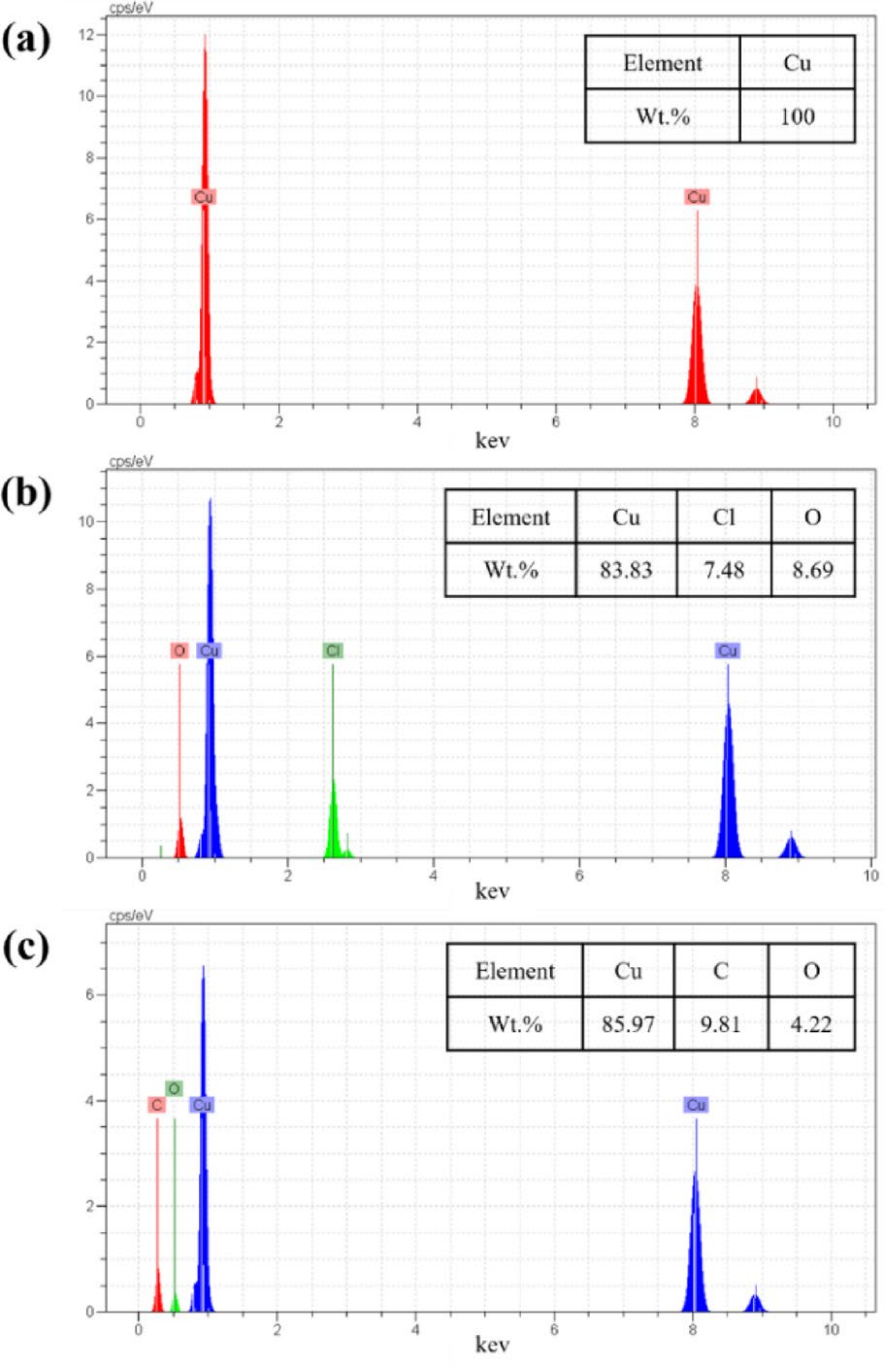
EDS diagrams obtained from the control Cu electrode **(a)**, the Cu electrode immersed in the corrosive solution of 3.5 wt% NaCl, in the absence **(b)** and presence **(c)** of the HBE inhibitor.

Furthermore, the graphs of EDS analysis reveal that the absence of the inhibitor in the corrosive solution leads to an increased percentage of elements such as Cl and O (Fig. 5b). The Cl originates from the NaCl present in the corrosive solution and contributes to surface attack. Additionally, the O in Fig. 5b reflects the formation of corrosion products on the surface. Conversely, Fig. 5c, which depicts the elemental analysis of the surface in the presence of the inhibitor solution, shows an increase in the percentage of C and O atoms, attributed to the presence of the inhibitor solution on the surface and the formation of a protective layer against corrosive agents.

### Thermodynamics studies

**Fig. 6:**
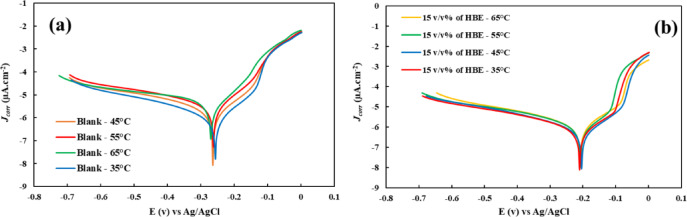
Tafel diagram investigating the corrosion of copper metal in a corrosive solution of 3.5 wt% NaCl in the presence **(b)** and absence **(a)** of optimum HBE concentration at temperatures of 308 to 338 K.

One of the most important applications of corrosion inhibitors is their use in high-temperature systems^42^. Additionally, due to the good thermal conductivity of copper, this metal is widely used in high-temperature pipelines, and therefore it is crucial to study the effects of temperature on the variation in the inhibitory efficiency of HBE. Consequently, the efficiency of this inhibitor was investigated at four temperatures ranging from 308 to 338 K. For this purpose, electrochemical potentiodynamic polarization analysis was performed at a scan rate of 5 mV. s^− 1^. This test was conducted in two sets of analyses; the first set examined the LSV diagrams of the copper electrode in a corrosive solution of 3.5 wt% NaCl without inhibitor at the mentioned temperatures (Fig. 6a), and the second set assessed the LSV diagrams in the presence of the optimal inhibitor concentration (15 v/v%) at these temperatures (Fig. 6b). The data obtained from these two series of tests are presented in Table 5. As observed, the IE% declines with increasing temperature, which could be attributed to the desorption of inhibitor molecules from the electrode surface due to the temperature rise.

**Table 5:** Electrochemical data extracted from Tafel plots of copper electrode in corrosive 3.5 wt% sodium chloride solution in the presence and absence of HBE.

	*T* (℃)	*E*_corr_ (mV. cm^−2^)	*j*_corr_ (µA. cm^−2^)	*-b*_c_ (mV. dec^−1^)	*b*_a_ (mV. dec^−1^)	*IE*%
**Blank**	35	−675.0	4.4	356.7	99.5	-
	45	−693.9	4.6	328.5	85.0	-
	55	−679.7	5.6	390.2	86.9	-
	65	−712.4	6.2	379.9	82.3	-
**HBE**	35	−553.4	2.4	345.6	36.8	45.5
	45	−532.9	2.5	365.0	73.7	45.7
	55	−541.3	2.7	348.8	57.8	51.8
	65	−535.8	3.1	323.6	93.5	50.0

According to the temperature study, the enthalpy (*ΔH*) and entropy (*ΔS*) of the corrosion reaction of copper metal in a corrosive NaCl solution in the presence of the HBE can be calculated using the transition-state equation obtained using the Arrhenius equation (Eq. 10):

10 $$\:\mathrm{ln}\frac{{j}_{corr}}{T}=\:\left[\mathrm{Ln}\left(\frac{R}{h{N}_{A}}\right)+\left(\frac{\varDelta\:S}{R}\right)\right]-\frac{\varDelta\:H}{R}\left(\frac{1}{T}\right)$$

**Fig. 7:**
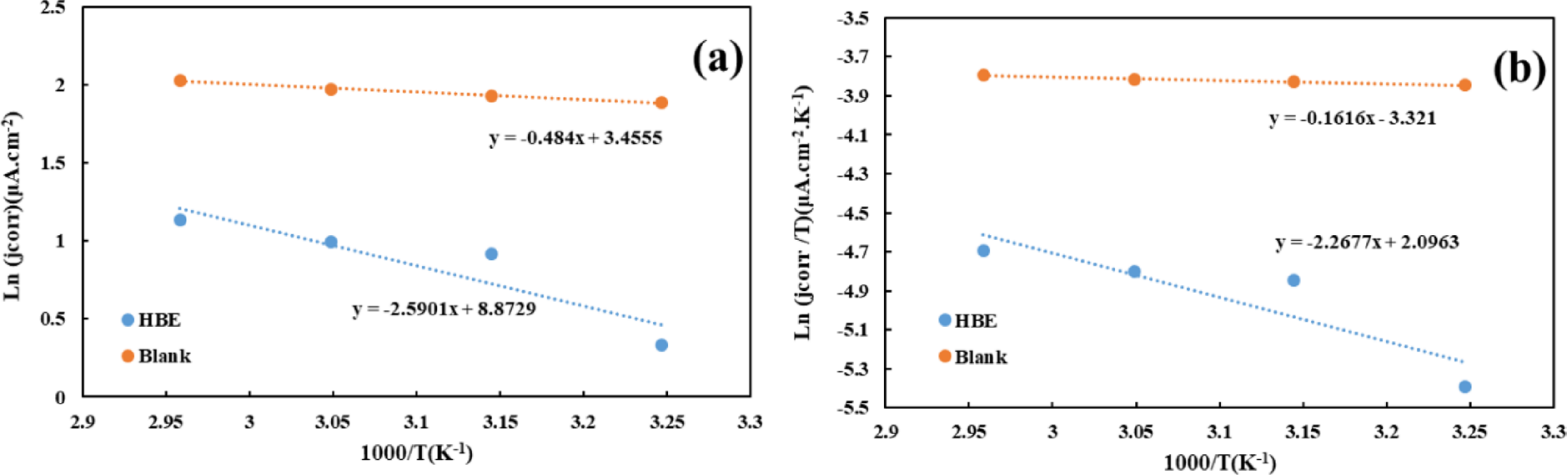
Arrhenius **(a)**, and Transition-state plots **(b)** of Cu in 3.5 wt% NaCl solution in the presence and absence of 15 v/v% of HBE.

Also, to examine the mechanism of inhibitor molecules adsorption on the surface of the copper electrode in the corrosive solution, ln(*j*_corr_) versus 1000/*T* can be drawn (Fig. 7a). The slope of this graph is -*E*_*a*_ *.R*^*− 1*^, which reports the activation energy (*E*_*a*_) of the corrosion reaction before and after using the HBE solution. The *E*_*a*_ of this reaction in the presence of the HBE increases from 4.1 to 21.5 KJ.mol^− 1^, which indicates that the corrosion reaction is more difficult to carry out from a thermodynamic point of view in the presence of the HBE. Also, if we plot $\:{ln}\frac{{j}_{corr}}{T}$ versus 1000/*T* (Fig. 7b), we can obtain Δ*H* and Δ*S* (Table 6). The slope of this plot is *ΔH*. *R*^− 1^ and the intercept is $\:\left[\mathrm{Ln}\left(\frac{R}{h{N}_{A}}\right)+\left(\frac{\varDelta\:S}{R}\right)\right]$. Positive Δ*H* indicates that the reaction is endothermic. However, the more negative Δ*S* with the addition of the HBE to the corrosive solution indicates a decrease in disorder due to the adsorption of inhibitor molecules on the electrode surface and a decrease in mass transfer to the active surface of the Cu electrode.

**Table 6:** Enthalpy and entropy calculated using the slope and intercept from the transition-state equation.

	ΔH (KJ. mol^−1^)	ΔS (KJ. mol^−1^. K^−1^)
Blank	1.34	−189.1
HBE	18.85	−199.2

### Adsorption isotherms

**Fig. 8:**
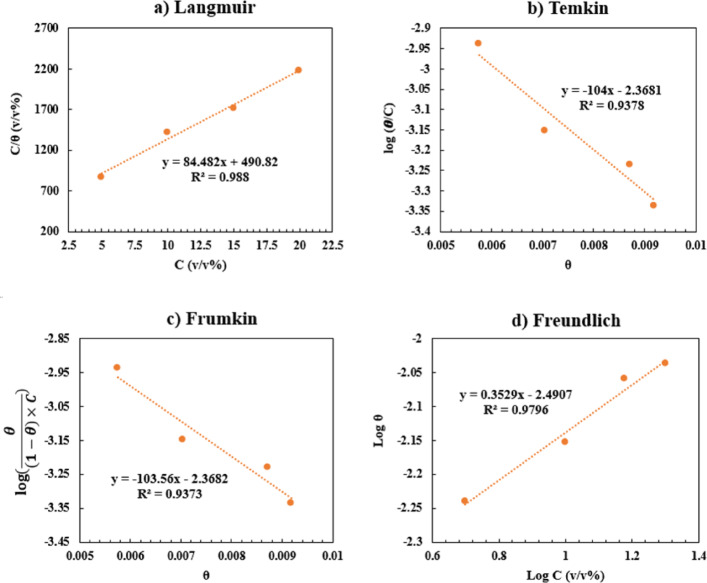
Langmuir **(a)**, Temkin **(b)**, Frumkin **(c)**, and Freundlich **(d)** isotherms for the adsorption of HBE molecules on the Cu electrode surface.

In order to investigate the mechanism of interaction of inhibitor molecules with the copper electrode surface, Langmuir, Temkin, Freundlich, and Framkin isotherms (Table 7) were plotted (Fig. 8).

**Table 7:** Equation of various isotherms of Langmuir, Temkin, Freundlich, and Frumkin.

Isotherm	Equation
Langmuir	$\:\frac{C}{\theta\:}=\:\frac{1}{{K}_{ads}}+C$
Frumkin	$\:\mathrm{\:log}\frac{\theta\:}{C}=\mathrm{log}{K}_{ads}-g\theta\:$
Temkin	$\:\mathrm{log}\left[\left(\frac{\theta\:}{\left(1-\theta\:\right)\times\:C}\right)\right]=2\alpha\:\theta\:+\mathrm{log}{K}_{ads}$
Freundlich	$\:\mathrm{log}\theta\:=\mathrm{log}{K}_{ads}+n\mathrm{log}C$

*C* represents inhibitor concentration

*K*_ads_ is the adsorption equilibrium constant

*θ* is related to the surface coverage

*n* is the Freundlich constant

*g* is the adsorption interaction parameter

As can be seen from the linear regression (*R*^*2*^) of each plot, the Langmuir isotherm has the closest *R*^*2*^ to 1 (Fig. 8a). Therefore, it can be concluded that the adsorption of inhibitor molecules follows the monolayer adsorption mechanism on the Cu electrode surface.

The adsorption behavior of HBE on the copper surface was analyzed using the Langmuir adsorption isotherm. A linear relationship was obtained from the C/θ versus C plot (Fig. 8a), confirming the applicability of the Langmuir model. The adsorption equilibrium constant (*K*_*ads*_), calculated from the intercept of the Langmuir plot, was found to be 2.04 × 10^− 3^ L. mol^− 1^.

The standard Gibbs free energy of adsorption (*ΔG*_*ads*_) was calculated using the relation *ΔG*_*ads*_ = − *RT* ln (55.5 *K*_*ads*_) and yielded a value of − 5.4 kJ. mol^− 1^, indicating that the adsorption of HBE on the copper surface is spontaneous and predominantly governed by physical adsorption. The deviation of the slope from unity may be attributed to surface heterogeneity and possible interactions among adsorbed species, which are commonly observed for plant extract inhibitors.

The relationship between the molecular constituents of *Hedge Bindweed* extract and their adsorption behavior on the copper surface was carefully analyzed. GC-MS/MS analysis revealed that the dominant compounds in the extract contain heteroatoms such as nitrogen, oxygen, and sulfur, along with polar functional groups including amide (-CONH-), hydroxyl (-OH), nitro (-NO_2_), amine (-NH_2_), and sulfide (-S-) moieties. These functionalities possess lone pair electrons and, in some cases, π-electron systems that can interact with vacant orbitals of surface copper atoms.

Adsorption occurs primarily through physical interactions, including electrostatic attraction between protonated inhibitor molecules and the negatively charged copper surface, as well as donor-acceptor interactions between heteroatom lone pairs and copper d-orbitals. This interpretation is consistent with the thermodynamic adsorption results, particularly the low and negative Gibbs free energy of adsorption (*ΔG*_*ads*_), indicating spontaneous and predominantly physisorption-controlled adsorption.

Furthermore, the adsorption behavior aligns with the experimentally observed Langmuir adsorption isotherm, suggesting monolayer formation without significant lateral interactions between adsorbed species. The gradual decrease in double-layer capacitance (*C*_*dl*_) observed in EIS measurements is attributed to the replacement of water molecules by adsorbed organic constituents of the extract, leading to increased interfacial thickness and a reduced dielectric constant. Overall, these findings establish a clear correlation between the chemical structures of the extract’s phytochemical components and their adsorption on the copper surface, providing a mechanistic understanding of the observed corrosion inhibition performance.

### Comparative analysis of copper corrosion inhibition by natural green extracts

Conventional corrosion inhibitors employed for copper protection, such as chromate-, nitrite-, and azole-based compounds, are widely recognized for their high inhibition efficiency; however, their application is increasingly restricted due to environmental toxicity, health hazards, and stringent regulatory limitations. These drawbacks have stimulated growing interest in the development of environmentally benign corrosion inhibitors derived from natural and renewable sources.

Based on the data presented in Table 8 and considering the overall scope of this study, the aqueous extract of *Hedge Bindweed* (Calystegia sepium, HBE) demonstrates a highly competitive, and in some cases superior, corrosion inhibition performance compared to several reported natural green inhibitors^3,22,43,44^. Inhibitors such as *turnip* peel extract (91.2%), *honeysuckle* extract (89.1%), and *Leonurus japonicus* (90.2%) exhibit notable inhibition efficiencies; however, HBE achieves the highest efficiency of 91.9% among the compared systems. This observation is fully consistent with the electrochemical and weight-loss results reported in the present work.

Importantly, while many reported green inhibitors require organic solvents or lack detailed extraction protocols, the excellent performance of *C. sepium* obtained using an aqueous extract indicates that its bioactive constituents are readily water-extractable and capable of forming a stable protective film on the copper surface. Similar behavior has been reported for other plant-based inhibitors operating in neutral or saline chloride environments, such as *Persea americana* and *Carpobrotus edulis* extracts, which showed high inhibition efficiencies for copper in NaCl solutions^43,45^.

Furthermore, the good thermal stability of HBE, maintaining approximately 50% inhibition efficiency at 65 °C, together with its conformity to the Langmuir adsorption isotherm, suggests a spontaneous adsorption process dominated by physical interactions. This adsorption behavior is consistent with the general inhibition mechanism proposed for phytochemical-rich green inhibitors containing heteroatoms capable of surface adsorption, as discussed in recent comprehensive reviews^46^.

Collectively, these findings indicate that *Hedge Bindweed* extract not only matches or exceeds the performance of several conventional and natural corrosion inhibitors, but also offers advantages in terms of environmental compatibility, adsorption behavior, thermal stability, and protective film formation, highlighting its strong potential as a sustainable and eco-friendly corrosion inhibitor for copper in saline environments.

**Table 8:** Comparison of several natural green inhibitors, the *IE%* of each inhibitor on copper metal, and the solvent extracting the inhibitor under study.

Corrosion inhibitor	Extraction solvent	IE (%)	Ref.
*Turnip* peel extract	water	91.2	^3^
*Honeysuckle* extract	water	89.1	^22^
*Carpobrotus edulis* leaf extract	-	87.3	^43^
*Leonurus japonicus Houtt*	water	90.2	^44^
HBE	water	91.9	This study

## Conclusion

This study highlights the potential of *Hedge Bindweed* Extract (HBE) as a sustainable and efficient green corrosion inhibitor for copper in 3.5 wt% NaCl solution. Beyond its strong inhibition performance, this work presents an innovative strategy to repurpose *Hedge Bindweed* an invasive and agriculturally problematic weed into a valuable industrial material. This transformation not only offers an eco-friendly alternative to synthetic inhibitors but also provides an environmentally responsible approach to weed management. Electrochemical analyses, including potentiodynamic polarization and electrochemical impedance spectroscopy (EIS), demonstrated the high inhibition efficiency of HBE (up to 91.9%), while weight loss tests revealed a comparable efficiency of 90.4%. Even at elevated temperatures (338 K), HBE retained satisfactory performance (50%), reflecting good thermal stability. Thermodynamic assessments indicated increased activation energy in the presence of the inhibitor, consistent with a hindered corrosion process. The positive enthalpy (ΔH) suggests endothermic adsorption, whereas the negative entropy (ΔS) indicates a more ordered inhibitor–metal interface. Surface characterization (SEM-EDS) further confirmed the formation of a protective organic layer that alleviated corrosion defects. Samples without HBE exhibited pronounced deterioration, whereas those with HBE displayed significantly smoother surfaces and lower levels of corrosive elements (Cl, O). These findings underscore the promise of HBE as a renewable, cost-effective, and eco-friendly corrosion inhibitor and contribute to advancing sustainable corrosion protection strategies for industrial applications.

## Supplementary Information

Below is the link to the electronic supplementary material.

Supplementary Material 1

## Data Availability

Data will be available on request.

## Abbreviations

GC-MS/MS: Gas-Chromatography/mas spectroscopy
EIS: Electrochemical impedance spectroscopy
LSV: linear sweep voltammetry
SEM: Scanning electron microscopy
EDS: Energy-dispersive X-ray spectroscopy, GPD, Gross Domestic Product
CMP: Chemical mechanical polishing
HBE: *Hedge bindweed* extract
