# Supplementary material for: Green corrosion protection of copper in chloride media with Calystegia sepium extract using electrochemical and GC-MS/MS analyses
Source: Sci Rep. 2026 Feb 27;16:11267. doi: 10.1038/s41598-026-41526-y (PMC13049151; doi:10.1038/s41598-026-41526-y)
Supplement: Supplementary file 1 — Supplementary Material 1 [file 41598_2026_41526_MOESM1_ESM.docx]

**Green corrosion protection of copper in chloride media with *Calystegia sepium* extract using electrochemical and GC-MS/MS analyses**

Mohammad Mahdi Alamnezhad^1^, Mehdi Hosseini^1,2*^, Mohammad Panahimehr^1,2^

*^1^Department of Chemistry, Faculty of Basic Sciences, Ayatollah Boroujerdi University, Boroujerd, Iran*

*^2^Biosensor and Energy Research Center, Ayatollah Boroujerdi University, Boroujerd, Iran.*

**Fig. S1.** GC-MS/MS spectrum of the ethanolic extract of *Hedge Bindweed* (HBE).
